# Computationally identifying hot spots in protein-DNA binding interfaces using an ensemble approach

**DOI:** 10.1186/s12859-020-03675-3

**Published:** 2020-09-17

**Authors:** Yuliang Pan, Shuigeng Zhou, Jihong Guan

**Affiliations:** 1 Department of Computer Science and Technology, Tongji University, No. 4800 Caoan Road, Shanghai, 201804 China; 2 Shanghai Key Laboratory of Intelligent Information Processing, and School of Computer Science, Fudan University, No. 220 Handan Road, Shanghai, 200433 China

**Keywords:** Protein-DNA complexes, Hot spots, Ensemble stacking classifier, Feature selection

## Abstract

**Background:**

Protein-DNA interaction governs a large number of cellular processes, and it can be altered by a small fraction of interface residues, i.e., the so-called *hot spots*, which account for most of the interface binding free energy. Accurate prediction of hot spots is critical to understand the principle of protein-DNA interactions. There are already some computational methods that can accurately and efficiently predict a large number of hot residues. However, the insufficiency of experimentally validated hot-spot residues in protein-DNA complexes and the low diversity of the employed features limit the performance of existing methods.

**Results:**

Here, we report a new computational method for effectively predicting hot spots in protein-DNA binding interfaces. This method, called *PreHots* (the abbreviation of *Pre*dicting *Hots*pots), adopts an ensemble stacking classifier that integrates different machine learning classifiers to generate a robust model with 19 features selected by a sequential backward feature selection algorithm. To this end, we constructed two new and reliable datasets (one benchmark for model training and one independent dataset for validation), which totally consist of 123 hot spots and 137 non-hot spots from 89 protein-DNA complexes. The data were manually collected from the literature and existing databases with a strict process of redundancy removal. Our method achieves a sensitivity of 0.813 and an AUC score of 0.868 in 10-fold cross-validation on the benchmark dataset, and a sensitivity of 0.818 and an AUC score of 0.820 on the independent test dataset. The results show that our approach outperforms the existing ones.

**Conclusions:**

*PreHots*, which is based on stack ensemble of boosting algorithms, can reliably predict hot spots at the protein-DNA binding interface on a large scale. Compared with the existing methods, *PreHots* can achieve better prediction performance. Both the webserver of *PreHots* and the datasets are freely available at: http://dmb.tongji.edu.cn/tools/PreHots/.

## Background

With the rapid development of structural biology technologies such as X-ray crystallography, NMR spectroscopy, and cryo-electron microscopy, a large number of tertiary structures of biological macromolecules have been generated [1]. However, the interpretation of these structures and the recognition of critical residues lie far behind the step of structure generation. Proteins and DNA are two kinds of most important biological macromolecules of life compounds. The interactions of proteins and DNA are essential for many crucial cellular processes, including gene expression and regulation, DNA replication and repair. For example, genes are regulated by the DNA-binding proteins that bind to some specific DNA sequences [2, 3]. Although DNA-protein binding interfaces contain a large number of residues, the associations between DNA and proteins are governed by a small fraction of residues with high binding affinity, which are also called *hot spots*. Hot spots are considered the most crucial residues for the formation and stabilization of protein complexes. Hence, accurate identification of hot spots is important to understand molecular regulation mechanisms and provide solutions to disease diagnosis and treatment [4].

At present, many experimental techniques have been used to measure protein-DNA binding free energy by site-directed mutagenesis, such as surface plasmon resonance (SPR) [5], isothermal titration calorimetry (ITC) [6] and fluorescence resonance energy transfer (FRET) [7]. However, these experimental techniques are not only inefficient and laborious, but also not suitable for dealing with the vast amounts of residues. Therefore, efficient and effective computational methods for identifying protein-DNA binding hot spots are greatly desirable and urgently needed.

Computational approaches can complement the experimental methods and make large-scale predictions efficiently. Molecular dynamics simulations and feature-based approaches are effective ways to identify hot spots. Two molecular dynamics simulation methods, SAMPDI [8] and PremPDI [9], were proposed to predict the change of protein-DNA binding free energy. SAMPDI utilizes the modified Molecular Mechanics Poisson-Boltzmann Surface Area (MM/PBSA) approach [10] along with additional knowledge-based features to predict binding affinity changes upon single mutation, while PremPDI relies on molecular mechanics force fields and fast side-chain optimization algorithms to evaluate the effects of single mutations on protein-DNA interactions. As for feature based approaches, a method called mCSN-NA [11] was developed, which uses graph-based signatures to predict the impact of a single mutation on protein-nucleic acid binding. Another feature-based approach PrPDH [12] was developed to predict protein-DNA binding hot spots. Although substantial advances have been made, there is still much space to explore for accurately identifying DNA-binding hot spots.

In this work, we develop a novel computational approach *PreHots* (the abbreviation of *Pre*dicting *Hots*pots), which is based on stack ensemble of boosting algorithms, for effectively predicting hot spots in protein-DNA binding interfaces. To this end, a dataset was constructed, which contains 260 samples from 89 protein-DNA complexes. More than half of the data are manually collected from the literature by ourselves, and the rest data are from the databases of ProNIT [13] and dbAMEPNI [14]. We totally calculated 157 features for fully representing hot spots, including not only the properties of the target residue but also target residues’ network information. From these features, a set of 19 informative features are selected by using a sequential backward selection algorithm. Extensive experiments were conducted on the benchmark dataset and the independent dataset to evaluate the proposed method, with comparison to existing methods. The experimental results show that our method can significantly boost the performance of DNA-binding hot spots prediction.

## Methods

**Fig. 1:**
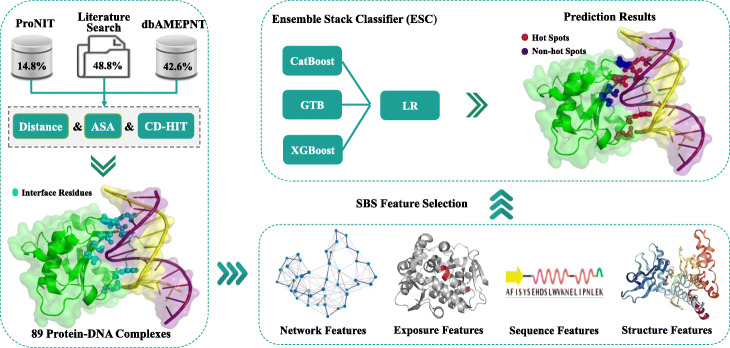
The workflow of *PreHots*

Figure 1 shows the workflow of the proposed method *PreHots*. First, a new reference dataset that consists of 123 hot spots and 137 non-hot spots from 89 protein-DNA complexes is constructed. The data are manually collecting from the literature and databases with a strict process of redundancy removal. Then, four types of features are encoded to characterize the target residues, including network features, exposure features, sequence features and structural features. Next, the informative features are selected by using sequential backward selection method. Following that, three boost classifiers, including categorical boosting (Catboost) [15], extreme gradient boosting (XGBoost) [16] and gradient tree boosting (GTB) [17] classifiers, are taken as the base models to form an ensemble stacking classifier (ESC), by a meta-model that adopts logistic regression (LR) [18] classifier. Finally, prediction results are output by the ESC model based on the selected feature set.

### Datasets

We constructed an initial dataset, containing experimentally measured binding free energy changes of 660 mutations from 162 protein-DNA complexes, which were obtained by combining two databases and manually searching the literature. Among them, 79 protein-DNA crystal structures were obtained from the database of ProNIT [13] and dbAMEPNI [14], and the other 83 protein-DNA crystal structures were manually collected from the literature.

To build high quality protein-DNA binding hot spots dataset, we used two methods to determine the interface residues. Solvent accessibility area (SAS) is widely used to identify interfacial residues, which can be obtained by calculating the difference of absolute solvent accessibility (*Δ*ASA >1Å) and the ratio of relative solvent accessibility (RASA >5%). And to make the results more accurate and stable, the ASA and RASA values of residues are calculated from protein structures by using Naccess [19]. Another method is to calculate the distance between the target residue and the DNA strand. If the distance is less than 5Å, the target residue can be considered as the interface residue. Moreover, we removed redundant homology sequences, where the similarity of protein sequences is more than 40% by using CD-HIT [20]. In this study, we define hot spots as the interface residues with the change in binding free energy (*ΔΔ*G) ≥1.0 kcal/mol, and the others are defined as non-hot spots. Finally, the constructed dataset consists of 123 hot spots and 137 non-hot spots from 89 complexes. In order to construct a balanced dataset to reduce the potential bias of the machine learning method, 64 protein-DNA complexes were randomly selected to form the benchmark dataset, which contains 90 hot spots and 90 non-hot spots. The rest of 25 protein-DNA complexes constitute the independent dataset, including 33 hot spots and 47 non-hot spots. To the best of our knowledge, our dataset is the largest one for predicting protein-DNA binding hot spots.

### Performance measures

We do performance evaluation by 10-fold cross-validation. The benchmark dataset is randomly divided into 10 subsets, each of which contains approximately the same number of samples. For each round, nine subsets are merged as the training set, while the remaining one subset is used for testing.

For comprehensively assessing the performance of our model, we adopted seven widely used evaluation metrics, including accuracy (ACC), sensitivity (SEN/Recall), specificity (SPE), precision (PRE), F1-score (F1), Matthew’s correlation coefficient (MCC) and the area under the ROC curve (AUC). ACC, SEN, SPE, PRE, F1 and MCC are defined as follows:

1 $$ SEN = \frac{TP}{TP + FN}  $$

2 $$ SPE = \frac{TN}{TN + FP}  $$

3 $$ PRE = \frac{TP}{TP + FP}  $$

4 $$ ACC = \frac{TP + TN}{TP + TN + FP + FN}  $$

5 $$ F1 = \frac{2 \times SEN \times Precision}{SEN + Precision}  $$

6 $$ MCC = \frac{TP \times TN - FP \times FN}{\sqrt{(TP + FP)(TP + FN)(TN + FP)(TN+FN)}}  $$

Above, *TP* is the number of true positives, *FP* is the number of false positives, *TN* is the number of true negatives and *FN* is the number of false negatives, respectively.

### Feature description

In order to explore informative features that play important role in the prediction of protein-DNA binding hot spots, we collected a comprehensive feature set that consists of 157 features, which can be roughly divided into four groups: residue interaction network features, solvent exposure features, and traditional features based on protein sequence and structure. More details about these feature are given below.

#### Residue interaction network features

As a representative kind of protein structures, residue interaction networks (RINs) have been widely and successfully used for revealing the effect of residues mutation, functional region and protein folding [21]. The traditional way to build RINs is to calculate the distance between *C*_*α*_ atoms of two residues within a certain threshold, which ranges from 5 to 9Å [22, 23]. But in fact, the interaction of protein-DNA depends on several intermolecular factors such as hydrogen bonds, van der Waals contacts, ionic bond and several other factors [24, 25]. The stability of protein-DNA interaction is maintained by forming hydrogen bonds between amino acid side chain residues of protein and DNA bases [26]. Therefore, the construction of RINs based on whether there is an intermolecular interaction between any two nodes, including residue and DNA, in the protein-DNA complexes by using RING [27]. In this study, five intermolecular interactions are considered: hydrogen bond, Van der Waals, disulfide bond, salt bridge, *π*- *π* stacking and *π*-cation.

To make the network contain more knowledge, each edge weight is assigned with the distance between two corresponding nodes. We calculate 10 RINs features that represent the importance of the target residue in the RINs, including node degree, clustering, closeness, betweenness, eigenvector, eccentricity, average neighbor degree, flow closeness, square clustering and Katz centrality.

#### Solvent exposure features

Solvent exposure of amino acid is crucial for exploring and predicting protein interaction and function. Solvent exposure features consist of several types of features, including half-sphere exposure (HSE), contact number (CN), residue depth (RD), accessible surface area (ASA) and relative accessible surface area (RASA). The solvent accessible has been extensively and successfully utilized to predict protein-protein interaction hot spots [28–31]. The limitation of solvent accessible is that it cannot provide any information about completely buried residues. Compared with traditional solvent accessible, half-sphere exposure (HSE) can describe the local environment of the target residue better from another perspective [32]. RD represents the average atom depth of target residue atoms, while CN is the number of residues in the sphere within a specific distance [33].

In this study, we calculated the characteristics of half-sphere exposure, contact number and residue depth, which could complement the solvent exposure information of interface residues. Based on protein sequence, a series of computing tools have been developed for predicting HSE, CN. We choose the method of HSEpred [32] and SPOT-1D [34] to calculate these features. For protein structure, we use hsexpo [33] to calculate the above three types of features, including HSE, CN and RD.

#### Structure-based features

Based on the three-dimensional structures of proteins, structure-based features were calculated, including hydrogen bonds, consensus scores, secondary structures, fluctuation score and solvent accessible surface area.

##### 1. Hydrogen bonds (Hbond).

The stability of protein-DNA interaction is maintained by forming hydrogen bonds between amino acid side chain residues of protein and DNA bases [26]. The hydrogen bond of protein-DNA complexes were calculated by using HBPLUS [35].

##### 2. Consensus scores.

Consensus score is a linear combination of residue interface propensity score, residue energy score and residue conservation score. Here, we used ENDES [36] to calculate consensus score, while the side chain energy score and relative solvent accessibility can also be obtained.

##### 3. Secondary structure (SS).

As an important feature, the secondary structural characteristics of residues were obtained from both sequences and structures of proteins. The definition of secondary structures of proteins (DSSP) [37] defines the secondary structure according to atomic coordinates in the protein data bank (PDB) [1]. In addition, several tools can predict the secondary structure of residue from protein sequence, including SPOT-1D [34], NetSurfp2 [38] and SPIDER3 [39].

##### 4. Fluctuation score.

The study of protein fluctuation is helpful to understand protein structures. FlexPred was used to predict the value of residue fluctuations [40]. Meanwhile, B-factor, represents the dynamic motion of atoms in a protein, was extracted from the PDB file.

##### 5. Solvent accessible surface area.

Solvent accessible surface area, including available surface area (ASA) and relatively accessible surface area (RASA), which has a strong correlation with hot spot prediction [12]. We applied Naccess [19] to calculate the ASA and RASA of residues from protein-DNA complexes.

#### Sequence-based features

Based on previous studies, we calculated many features of protein-DNA binding residues from protein sequences.

##### 1. Position-specific scoring matrix (PSSM).

It is well known that PSSM is an essential feature for predicting hot spots [4, 28, 31]. PSSM score represents the relationship between the frequency of amino acid substitutions and that expected by chance. Negative numbers indicate less frequent substitutions than expected by chance, while positive numbers mean more frequent substitutions than expected.

##### 2. Conservation score.

Conservative analysis of residues is extensively used to identify functionally important residues in protein sequences. The conservation score of residues can be calculated by using Jensen-Shannon divergence [41].

##### 3. Solvent accessible surface area.

Apart from deriving solvent accessibility from protein structure, we also used SPIDER3 [39] and NetSurfp2 [38] to calculating ASA and RASA from protein sequence.

##### 4. Physicochemical features.

Amino acid indices database (AAindex) collects various biochemical and physicochemical characteristics of amino acids [42]. In this work, protein-DNA binding hot spots are described by eight physicochemical characteristics: propensities, polarity, hydrophilicity, average accessible surface area, atom-based hydrophobic moment, flexibility parameter for no rigid neighbors, hydrophobicity and polarizability.

##### 5. Blocks substitution matrix (BLOSUM).

BLOSUM62 [43] means that sequence similarity is more than 62% in terms of sequence alignment. We calculated BLOSUM62, the most widely used amino acid scoring matrix, whose scores indicate the similarity between two types of amino acids.

##### 6. Local structural entropy (LSE).

Previous research found that local structural entropy is related to the stability of protein, and it was successfully used for predicting protein-protein interaction hot spots. In this work, we calculated the LSE [44] value of each residue within a protein sequence.

##### 7. Disordered regions (DISO).

Recognizing protein disorder regions contributes to the understanding of protein function and protein fold pathway. SPOT-Disorder [45] and RaporX-Property [46] were used to predict disorder regions of protein-DNA binding residues.

### Feature selection

For high-dimensional datasets, feature selection can effectively remove some irrelevant features, which contributes to lifting the efficiency of learning tasks and making the model easier to be understood. We used a sequential backward selection (SBS) algorithm to select a subset of informative features that are highly relevant to protein-DNA binding hot spots from the initial set of 157 features. Sequential backward selection (SBS), which is a heuristic search algorithm, removes one feature each time till an optimal feature subset is generated. Here, each resulting feature set is evaluated by using 10-fold cross-validation with the ESC classifier. Such 10-fold cross-validation procedure is repeated 30 times and the average performance over 30 trials is taken as the result. Besides, we combine the independent dataset and each cross-validation test dataset as the test dataset, which is used to evaluate features and obtain the evaluation score at each 10-fold cross-validation. The evaluation metric of feature selection is represented by *E*_*c*_, calculated as follows:

7 $$ E_{c}\,=\,\frac{1}{R}\!\sum_{R=1}^{R}\!\left\{\frac{1}{n}\sum_{n=1}^{n}\left({ACC}_{i} \!+ \!{SEN}_{i}\! + \! {SPE}_{i}\! +\! {MCC}_{i}\! + \!{AUC}_{i}\!\right)\!\right\}  $$

where *R* is the number of cross-validation; *n* is the number of iterations of 10-fold cross-validation; *ACC*_*i*_, *SEN*_*i*_, *SPE*_*i*_, *MCC*_*i*_, and *AUC*_*i*_ indicate the values of accuracy, sensitivity, specificity, Matthew’s correlation coefficient and AUC score of the *i*-th 10-fold cross-validation, respectively.

In the SBS method, features are iteratively removed one by one from the initial feature set. In the first round, each feature is deleted once (resulting in 157 subsets of 156 features). If the ESC classifier based on a certain feature subset achieves the higher *E*_*c*_, this feature subset is left for the next round of feature selection. Such a feature selection process would continues till *E*_*c*_ does not increase any more.

### Ensemble stacking classifier

Stacking, also called super learning [47], is an ensemble machine learning method that constructs the base-level models and meta-model by combining different machine learning classifiers. The construction of base-level models is based on the benchmark dataset, and the meta-model is trained on the outputs of the base-level models. The ensemble stacking classifier (ESC) can overcome the disadvantage of single classifier and make the prediction more robust than a single model. In this study, we choose three boost classifiers as the base-level models, which are categorical boosting (Catboost) [15], extreme gradient boosting (XGBoost) [16] and gradient tree boosting (GTB) [17] classifiers, and the meta-model adopts logistic regression (LR) [18] classifier.

## Results and discussion

### Performance of the ensemble stacking classifier

Ensemble stacking classifier (ESC) is an ensemble technique that the output of the first-level (base) classifiers is taken as the input of the second-level classifier by constructing a two-level model. In this study, the first-level classifiers consist of categorical boosting (Catboost) [15], extreme gradient boosting (XGBoost) [16] and gradient tree boosting (GTB) [17] models, and the second-level classifier is a logistic regression (LR) [18] model. To check whether ESC is suitable for predicting hot spots in the complexes, we compared ESC with ensemble vote classifier (EVC) and some popular machine learning models, including random forests (RF) [48], GTB, support vector machine (SVM) [49], Catboost and XGBoost. Among them, the ensemble vote classifier (EVC) is another ensemble technique, which integrates different machine learning algorithms and predicts hot spots by using the average predictive probability of all algorithms. To avoid the randomness of cross-validation results, we do 10-fold cross-validation 30 times and the averaged result of all 30 cross-validation trials is taken as the final result. Table 1 shows the results of ESC and the compared methods. We can see that the ensemble techniques are generally superior to the other machine learning methods. And, ECS outperforms EVC and can significantly improves the performance of hot- spots prediction.

**Table 1:** Performance comparison between ESC and five existing classifiers

Method	ACC	SEN	SPE	PRE	F1	MCC	AUC
RF	0.683	0.696	0.684	0.687	0.669	0.374	0.758
SVM	0.685	0.673	0.695	0.670	0.665	0.366	0.793
CatBoost	0.722	0.731	0.726	0.734	0.721	0.455	0.806
GTB	0.711	0.743	0.733	0.718	0.705	0.468	0.816
EVC	0.725	0.741	0.721	0.699	0.694	0.446	0.826
ECS	0.783	0.795	0.753	0.784	0.782	0.562	0.833

### Performance of feature selection

**Fig. 2:**
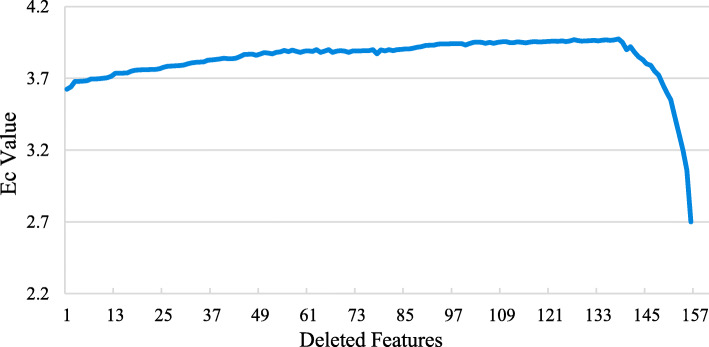
The change of *E*_*c*_ value in the process of stepwise feature selection

Feature selection is crucial for building accurate classification models, which aims to select a small number of informative features. In this study, our initial feature set consists of 157 candidate features, which can be divided into four groups: residue contact network features (network), solvent exposure features (exposure), sequence features and structural features. We used a sequential backward selection (SBS) method to choose relevant and informative features from the initial feature set. SBS uses a stepwise feature selection scheme, which iteratively removes features one by one from the feature set. The evaluation criterion (*E*_*c*_) represents the average prediction performance of ESC when selecting features. Figure 2 shows how *E*_*c*_ changes during the process of stepwise feature selection. *E*_*c*_ reaches the maximum when the number of selected features is 19. Consequently, these 19 features form our optimal feature set.

To assess the advantage of the SBS method, we compared it with four existing methods, including random forest (RF), recursive feature elimination (RFE) [50], maximum relevance minimum redundancy (mRMR) [51] and the block Hilbert-Schmidt independence criterion (HSIC) Lasso [52]. The commonly used methods are RF, RFE and mRMR, which use the mean decrease Gini index (MDGI), SVM-based recursive feature elimination and max relevance and min redundancy criteria to evaluate the importance of features, respectively. The block HSIC Lasso (HSIC Lasso) is a relatively novel method, which adopts an effective nonlinear feature selection algorithm based on HSIC Lasso to select informative biological features. To obtain reliable results, we ran 30 times of 10-fold cross-validation and took the average performance as final result. Table 2 shows the performance of the five feature selection methods on the benchmark dataset. We can see that SBS can select better features, which are helpful to predict protein-DNA binding hot spots. And the ESC classifier with SBS achieves the best prediction performance, with a 0.535 MCC and a 0.853 AUC.

**Table 2:** Performance comparison between SBS and four existing feature selection methods

Method	ACC	SEN	SPE	PRE	F1	MCC	AUC
RF (28)	0.744	0.739	0.749	0.716	0.715	0.483	0.823
RFE (20)	0.739	0.723	0.730	0.719	0.718	0.452	0.830
mRMR (25)	0.755	0.787	0.746	0.766	0.761	0.531	0.835
HSIC Lasso (30)	0.740	0.777	0.727	0.746	0.744	0.500	0.841
SBS (19)	0.767	0.784	0.766	0.776	0.741	0.535	0.853

### Significance of selected features

**Fig. 3:**
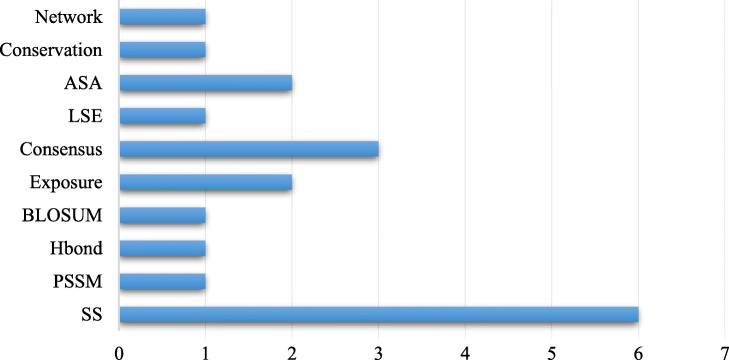
The number of each class features on the optimal feature set

By using the SBS feature selection method, we obtain an optimal feature set, which contains 19 features as shown in Table 3. The ranking of these selected features is based on F-score, which is to measure the distinguishing ability of features between hot and non-hot spots. The most important features include PSSM, hydrogen bonds, secondary structure and RINs features. Two exposure features (as novel features) are selected into the optimal feature set, which indicates that they are important features for identifying DNA-binding hot spots. Fig. 3 shows more details about the distribution of selected features in different feature categories. Six secondary structural features are selected. In previous works, secondary structural has been considered as a fundamental and essential features to improve prediction performance. In this work, we derived secondary structural features from two levels of protein structures and sequences, which can provide a more comprehensive description of secondary structural characteristics of target residues. Besides, ASA, exposure features and consensus score also contribute significantly to the prediction of hot spot residues. These results suggest that the ten categories of 19 optimal features can complement each other and accurately describe the hot spot residues, thus collectively improve the prediction performance.

**Table 3:** The rankings of the 19 selected features

**Rank**	**Feature name**	**Type**	**Rank**	**Feature name**	**Type**
1	PSSM(R)	Sequence	11	Lse score	Sequence
2	H-Bond in HBPLUS	Structure	12	phi in SPOT-1D	Sequence
3	ALPHA in Xssp	Structure	13	COMBINED2 score in ENDES	Structure
4	Current_flow_closeness_centrality	Network	14	ACC in Xssp	Structure
5	Q3_prob_3 in NetSurfp2	Sequence	15	RSA in NetSurfp2	Sequence
6	HSEa-u in SPOT-1D	Exposure	16	P(8-G) in SPOT-1D	Sequence
7	COMBINED1 score in ENDES	Structure	17	CN in hsexpo	Exposure
8	SIDESCORE score in ENDES	Structure	18	Conservation score	Sequence
9	Q8_prob_1 in NetSurfp2	Sequence	19	Blosum(E)	Sequence
10	P(8-I) in SPOT-1D	Sequence			

These features fall intro four types, i.e., network features, exposure features, structure features and sequence features

### Performance comparison with state-of-the-art methods

Here, we further compare our method with four existing protein-DNA binding hot spots prediction methods, including PrPDH [12], PremPDI [9], mCSM-NA [11] and SAMPDI [8], on the benchmark dataset and the independent test dataset. PrPDH uses a classification model to identify hot-spots from various interface residues, while PremPDI, mCSM-NA and SAMPDI use regression models to predict the change of Protein-DNA binding free energy.

Table 4 presents the results on the benchmark dataset, where the prediction results of existing methods are from their websites. In general, our method performs better than the other methods in terms of six of the seven metrics (ACC, SEN, SPE, FRE, F1, MCC and AUC). Only our SPE is smaller than that of the mCSM-NA method.

**Table 4:** Performance comparison between our method with four existing methods on the benchmark dataset

Method	ACC	SEN	SPE	PRE	F1	MCC	AUC
PreHots	0.789	0.813	0.801	0.785	0.784	0.597	0.868
PrPDH	0.683	0.667	0.700	0.690	0.678	0.367	0.779
PremPDI	0.756	0.711	0.800	0.780	0.744	0.513	0.790
mCSM-NA	0.461	0.056	0.867	0.284	0.093	-0.133	0.314
SAMPDI	0.544	0.444	0.644	0.556	0.494	0.091	0.522

Table 5 gives the results on the independent test dataset. Compared with the existing methods, our method significantly improves the prediction performance. Concretely, 81.8% of the true hot spots are correctly predicted (SEN = 0.818) and 76.6% of the non-hot spots are correctly predicted (SPE = 0.766). Except for SPE, our method achieves the highest values of the other metrics, especially for the comprehensive indexes MCC (0.576) and AUC (0.82). These results show that our method is superior to the existing methods in identifying protein-DNA binding hot spots.

**Table 5:** Performance comparison between our method with four existing methods on the independent dataset

Method	ACC	SEN	SPE	PRE	F1	MCC	AUC
PreHots	0.788	0.818	0.766	0.711	0.761	0.576	0.820
PrPDH	0.600	0.545	0.638	0.514	0.529	0.182	0.628
PremPDI	0.463	0.333	0.553	0.344	0.338	-0.114	0.411
mCSM-NA	0.563	0.121	0.872	0.400	0.186	-0.010	0.472
SAMPDI	0.545	0.272	0.727	0.400	0.324	0.000	0.525

### Case study

#### The *λ* exonuclease (*λ*exo) and DNA complex.

**Fig. 4:**
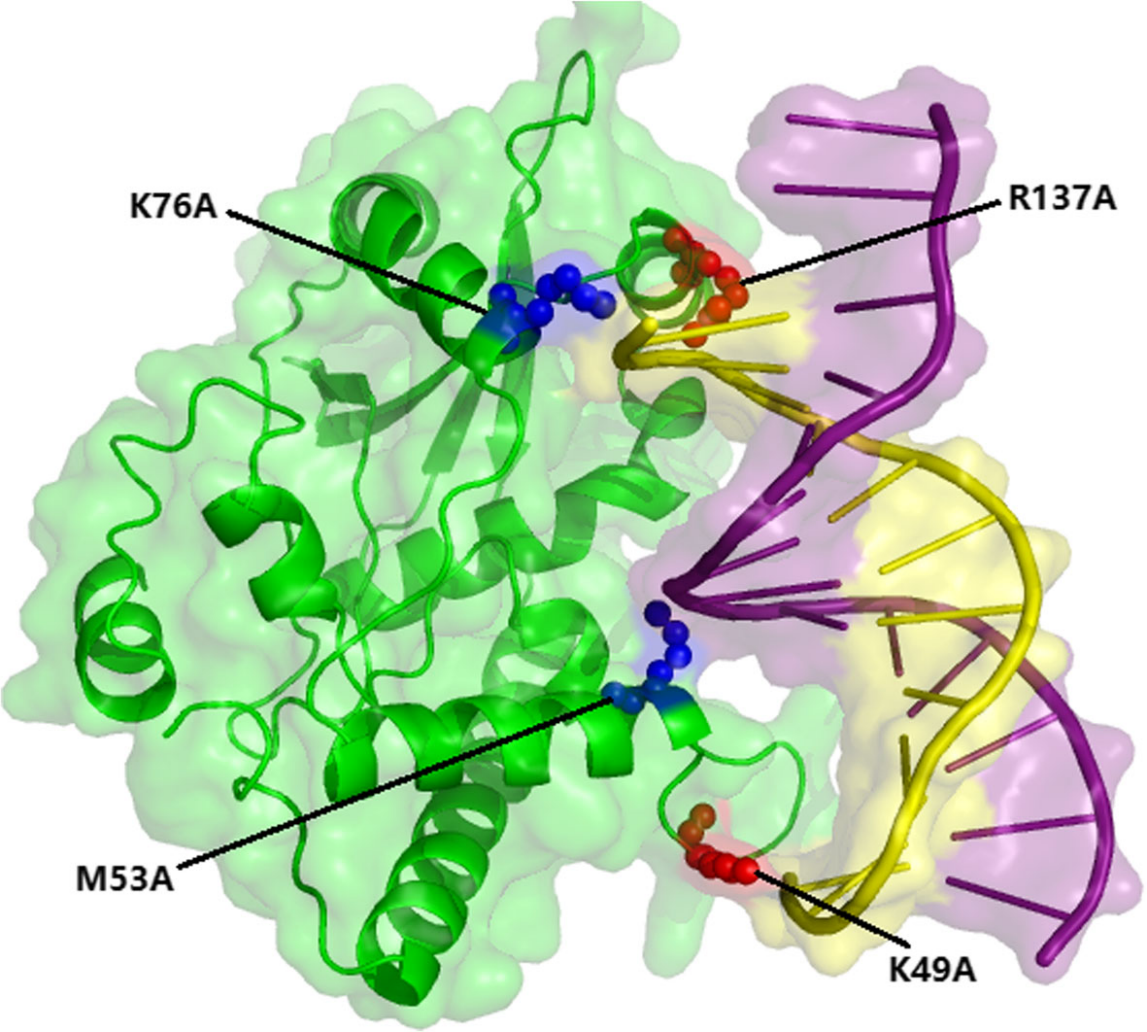
The hot spot residues of *λ*exo-DNA complex (PDB ID: 3SM4) identified by experiments. The green surface denotes the protein chain (chain A) while the purple and yellow surfaces represent the DNA chains (purple: chain E and yellow: chain D). The red color represents experimentally identified hot spot residues and the blue color represents experimentally determined non-hot spot residues

*λ*exo is an ATP-independent enzyme that binds double-stranded DNA (dsDNA) to form the *λ*exo-DNA complex (PDB ID: 3SM4, chain: A) [53]. Four mutated interfacial residues of the *λ*exo-DNA complex have experimentally been identified and shown in Fig. 4. The hot spots residues (*ΔΔ*G) ≥1.0 kcal/mol are K49_A and R137_A, and the rest are non-hot spots (K76_A and M53_A). Our approach successfully identified all the hot spots, while only a non-hot spot (K76_A) was wrongly identified. In addition, PremPDI, PrPDH and SAMPDI only correctly predicted two non-hot spots (K76_A and M53_A), while the two hot spots were wrongly predicted. mCSM-NA only correctly predicted one non-hot spots (M53_A). This example shows that our method can effectively identify hot spots from protein-DNA complexes than the major existing methods.

#### DNA-bound SUP- 12_28−121_ complex.

**Fig. 5:**
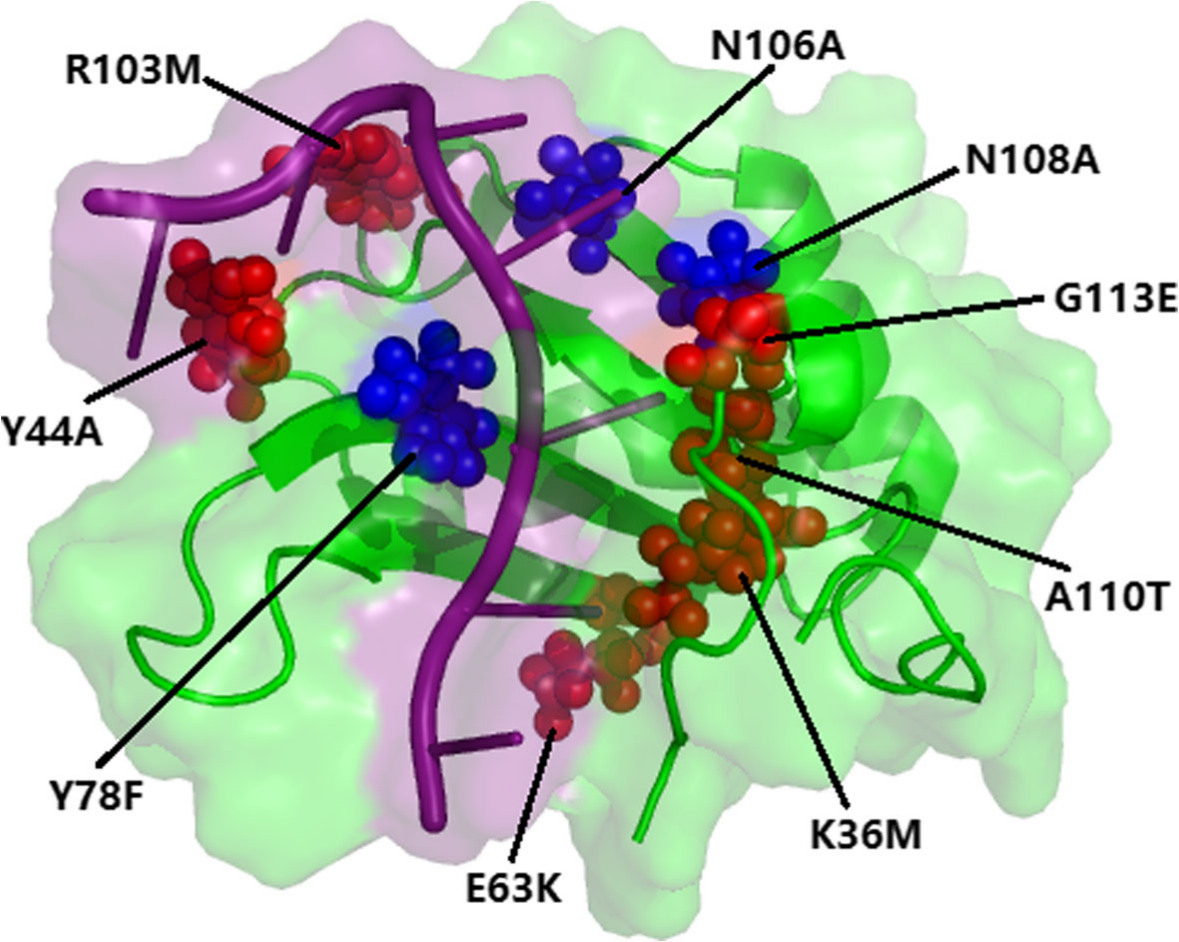
The hot spot residues of DNA-bound SUP- 12_28−121_ complex (PDB ID: 4CH1) identified by experiments. The green surface denotes the protein chain (chain A) while the purple surface represents the DNA chain (chain B). The red color represents experimentally identified hot spot residues and the blue color represents experimentally determined non-hot spot residues

The structure of DNA-bound SUP- 12_28−121_ (PDB ID:4CH1, chain: A) can provide accurate clue to the mechanism of DNA recognition [54]. The defined hot spot residues are K36_M, Y44_A, E63_K, R103_M, A110_T and G113_E, and the remaining three residues (Y78_F, N106_A and N108_A) are non-hot spots (see Fig. 5). For these nine mutated residues, PremPDI identified three of the six hot spots (K36_M, R103_M and A110_T) and one non-hot spot (Y78_F). PrPDH predicted two residues as hot spots (R103_M and A110_T) and the others as non-hot spots. SAMPDI identified one residue as hot spot (Y44_A) and the others non-hot spots, while mCSM-NA predicted all residues as non-hot spots. On the contrary, except for a hot spot (K36_M), our method predicted correctly all the other residues. This suggests that our method has the highest accuracy, which is desirable for many biological applications.

### Webserver

A user-friendly webserver of *PreHots* has been implemented, which is available at: http://dmb.tongji.edu.cn/tools/PreHots/. The input to *PreHots* should be the PDB file, which contains at least one protein chain and one DNA strand. The user can select the chain of protein and DNA, and submit the job to the server. Then, *PreHots* will return a list of residues, which are predicted to be potential DNA-binding hot spots based on the ensemble classifier with optimally features. Interface residues are colored according to the predicted confidence score. For visual display, users can use the 3D viewer to display prediction results and download the results. Multiple PDB files can be submitted simultaneously, and the jobs are executed in parallel on a cluster server with multiple computing nodes to lift prediction efficiency.

## Conclusion

Computational approaches can effectively and efficiently distinguish hot spots and non-hot spots from protein-DNA complexes on a large scale. In this work, we present a new computational method named *PreHots* for predicting hot spots in protein-DNA complexes. Compared with the existing methods, *PreHots* uses a high-quality dataset manually curated from literature and databases and with a strict process of redundancy removal. A large number of related features (network, exposure, sequence and structure) were calculated to characterize the residues from various aspects. To improve prediction performance, we used the SBS feature selection method to get the optimal feature set and constructed the classification model by the ESC method that integrates four well-performing models. Our method overcomes the drawbacks of single classifiers and makes the prediction more robust. We conducted extensive experiments to evaluate the proposed method, and compared it with existing methods on both a benchmark dataset and an independent test dataset. Experimental results show that our approach achieves higher overall performance than the existing methods. We believe that our method is an invaluable tool of identifying hot spot residues in protein-DNA complexes and can provide insights for the characterization of protein-DNA binding sites.

## Data Availability

*PreHots* is free available at http://dmb.tongji.edu.cn/tools/PreHots/.

## Abbreviations

PreHots: The abbreviation of predicting hot spots
SPR: Surface plasmon resonance
ITC: Isothermal titration calorimetry
MM/PBSA: Molecular mechanics poisson-boltzmann surface area
Catboost: Categorical boosting
XGBoost: Extreme gradient boosting
GTB: Gradient tree boosting
ESC: Ensemble stacking classifier
LR: Logistic regression
SAS: Solvent accessibility area
RASA: Relative solvent accessibility area
ACC: Accuracy
SEN: Sensitivity
SPE: Specificity
PRE: Precision
F1: F1-score
MCC: Matthews correlation coefficient
AUC: Area under curve
HSE: Half-sphere exposure
CN: Contact number
RD: Residue depth
Hbond: Hydrogen bonds
SS: Secondary structure
PDB: Protein data bank
PSSM: Position-specific scoring matrix
AAindex: Amino acid indices database
BLOSUM: Blocks substitution matrix
LSE: Local structural entropy
DISO: Disordered regions
SBS: Sequential backward selection
ESC: Ensemble stacking classifier
EVC: Ensemble vote classifier
RF: Random forests
SVM: Support vector machine
RFE: Recursive feature elimination
mRMR: Maximum relevance minimum redundancy
HSIC: Hilbert Schmidt independence criterion
MDGI: The mean decrease Gini index
FRET: Fluorescence resonance energy transfer
